# DIC Score: Statistical Relationship with PT, APTT, and Simplified Scoring Systems with Combinations of PT and APTT

**DOI:** 10.5402/2012/579420

**Published:** 2012-03-05

**Authors:** Vani Chandrashekar

**Affiliations:** Department of Hematology, Apollo Hospitals, 21, Greams Lane, Off Greams Road, Chennai, Tamil Nadu 600006, India

## Abstract

We looked into the statistical association of prothrombin time (PT) and activated partial thromboplastin time (APTT) with disseminated intravascular coagulation (DIC) score calculated using the International society for thrombosis and haemostasis (ISTH) scoring system. The PT, APTT, PT + APTT, and PT/APTT ratios were evaluated against the DIC score by linear regression analysis in fifty inpatients with suspected DIC. The PT, PT + APTT, and PT/APTT ratios were all found to be statistically significant in predicting DIC scores with *P* values of 0.02, 0.03, and 0.02, respectively. The APTT alone was not found to be statistically significant in predicting DIC score and had a *P* value of 0.09. This scoring system does not need d-dimer levels and the platelet count.

## 1. Introduction

Disseminated intravascular coagulation (DIC) is characterised by intravascular fibrin formation and disturbance of the microvasculature [1]. It is a complex disorder with multiple interactions involving hemostasis, fibrinolysis, and inflammation. The International Society for Thrombosis and Haemostasis proposed a definition and a scoring system for DIC [2]. This scoring system takes into account prothrombin time (PT), fibrinogen levels, levels of fibrin-related marker and platelet count.

The PT is usually abnormal in DIC but may be normal and hence has been termed as an unreliable test in this setting [3–6]. PT is prolonged in 50–75% of patients with DIC, and in 25–50% it is normal or shortened [7]. Activated partial thromboplastin time (APTT) is prolonged in 50–60% of patients with DIC, but a normal or shortened APTT may also be seen [7].

 A biphasic clot waveform of APTT has been reported by some observers as an early event in DIC and noted to have an adverse outcome by others [8, 9].

The statistical relationship, if any between DIC scores and PT and APTT, has not been previously reported in the literature. We undertook this study to evaluate this relationship in fifty patients with underlying conditions associated with DIC.

## 2. Materials and Methods

We evaluated blood samples from fifty patients (from February 2011 to June 2011) with underlying condition for DIC and were clinically suspected of having DIC. All these patients had bleeding tendencies. No thrombotic episodes were reported for these patients. None of them were known to be on anticoagulants or had any prior bleeding tendencies. Samples were evaluated at the time of admission.

Blood samples were collected in 3.2 gm% trisodium citrate as anticoagulant (9 : 1 ratio) and immediately centrifuged at 2500 g for fifteen minutes. The PT (Thromborel S reagent, Siemens), APTT (Dade Actin FSL Activated PTT reagent, Siemens), fibrinogen (STA-Fibrinogen reagent), and d-dimer (STA-LIATEST D-DI) were evaluated on the STA compact analyser. Platelet counts were obtained on the ADVIA 2120 analyser using EDTA anticoagulated samples. Control samples were prepared from twenty healthy individuals (equal number of men and women) who were not on any anticoagulants.

A DIC score was calculated using the ISTH scoring system. Platelet count more than 100 × 10^9^ per liter was given a score of 0, between 50–100 × 10^9^ per liter score of 1 and less than 50 × 10^9^ per liter a score of 2. Elevated fibrin-related marker was given the score 0 if there was no increase, 2 if there was moderate increase, and 3 if there was strong increase. A prolonged PT of less than 3 seconds was given a score of 0, more than 3 and less than 6 was given a score of 1, and more than 6 was given a score of 2. Fibrinogen levels below 1 g/L were given a score of 0, and levels above 1 g/L were given a score of 1. The DIC score was calculated by adding all these values for the four parameters.

 Linear regression analysis with the DIC score as dependant variable on the *y*-axis and APTT as independent variable on the *x*-axis was plotted using Microsoft Excel Analysis pak tool kit. PT, the sum of PT + APTT, and the ratio of PT/APTT as independent variables on the *x*-axis were plotted likewise against DIC scores on the *y*-axis. The ANOVA tables were obtained and *P* values (significant *P* value less than 0.05) were calculated. With the regression coefficient and the intercept, predicted DIC scores were calculated for the fifty samples using the equation *y* = *a* + *bx*, where *y* is the dependant variable (DIC score), *a* is the intercept or slope, *b* is the regression coefficient, and *x* is the independent variable. 

## 3. Observations

Among these fifty patients, there were thirty-eight males and twelve females. They were in the age group of 30–81 years. Sepsis was the commonest underlying condition for DIC (Table 1).

 The PT was elevated in 46 patients. The difference of PT from the control varied from 1–169 seconds.

The APTT was elevated in 41 patients. The difference of APTT from the control varied from 2–152 seconds.

Both PT and APTT were together elevated in 41 patients.

An ISTH DIC score of 0 was seen in three of our patients, score of 1 in two patients, score of 2 in seven patients, score of 3 in eight patients, score of 4 in sixteen patients, score of 5 in eight patients, and score of 6 in six patients.

The APTT alone was not found to be statistically significant (*P* value of 0.09) when plotted against DIC scores (Table 2).

The PT/APTT ratio was found to be statistically significant (*P* value of 0.020) in predicting DIC score (Table 3). Likewise, PT and PT + APTT were also found to be statistically significant (*P* values of 0.02 and 0.03, resp.) (Tables 4 and 5).

The mean predicted DIC score (Tables 6, 7, 8, 9, 10, 11, and 12) using PT alone increased from 3.35 to 4.10 for the ISTH scores of 0–6 except for the ISTH score of 2 where it was 3.38 which was less than the ISTH score of 1 where it was 3.42 and score 5 (3.57) which was less than the score of 4 (3.60). The mean score using PT + APTT increased from 3.32–3.93 except for the score of 2 (3.35) which was less than the score of 1 (3.37). The mean score using PT/APTT ratio also increased from 3.31–3.92 except for the score of 2 (3.37) which was less than the score of 1 (3.58).

## 4. Discussion

In an appropriate clinical setting DIC is diagnosed with an elevated PT, APTT, low platelets, and elevated d-dimer levels. However, PT and APTT are not always elevated, and both normal and shortened times have been reported and as such they have been found to be unreliable [3–7]. The d-dimer assay appears to be the most reliable test [7].

 In our case series, we proceeded to evaluate the statistical association of PT and APTT with the DIC scores calculated using ISTH scoring system. Most of our patients had elevated PT and APTT. None of our patients had a shortened PT or APTT. The APTT alone did not have statistical significance with the DIC scores. However, PT, PT + APTT, and PT/APTT ratios were found to be statistically significant with *P* values of 0.02, 0.03, and 0.02, respectively.

As the ISTH scores increased from 0 to 6, our predicted DIC scores also increased from 3.31 to 3.92 (PT/APTT ratio), 3.32–3.93 (PT + APTT) except for the ISTH score of 1 where our predicted score was 3.58 (PT/APTT), 3.37 (PT + APTT) compared to the ISTH score of 2 where our predicted score was 3.37 (PT/APTT), 3.35 (PT + APTT). There were only two patients with the ISTH score of 1, one of whom had minimally elevated PT which would be given a score of 0 in the ISTH system (as PT elevation of less than 3 seconds were given a score of 0). However, our system is sensitive to even minor elevations in PT, making an impact on the predicted score. The other patient had elevated PT (6 seconds above control value), but normal platelet count and minimally elevated d-dimer. With all other scores as the ISTH score rose, our mean predicted DIC score also increased. Using PT alone, our predicted score rose comparably except for the scores of 1 and 5. Like other scoring systems for DIC, our system is only relevant in patients who have an underlying condition associated with DIC. Existing undetected factor deficiencies such as Hageman factor which are not clinically significant will have profound effect as APTT will be elevated. Though our study included many patients with liver disease, only those patients with elevated d-dimer, where DIC had set in were included in the study.

Our paper does not refute the importance of tests for d-dimer, prothrombin fragment 1 + 2, thrombin antithrombin complex, plasmin antiplasmin complex, decreased antithrombin III, and other tests used for diagnosing DIC. Nor does it imply that the scoring systems such as ISTH can be replaced. We hope this paper can identify groups of patients who may benefit from replacement therapies (such as fresh frozen plasma or recombinant factor viia when the PT/APTT or PT + APTT is rising and withholding fresh frozen plasma and administration of heparin when these values are on the decline) and also monitor their efficacy with repeated calculation of the predicted DIC scores. It might be argued that a simple elevated PT and APTT can guide therapy, and there is no need for calculation of the DIC score. However, minor PT elevations of 1–3 seconds are ignored and these patients do have underlying conditions for development of DIC and they need to be monitored. Another scenario where our study could come of use is when there is a shortened APTT which also tends to be ignored in the ISTH scoring system. This could also be of great help in hospitals with limited facilities where a d-dimer test is not feasible. Platelet counts on analysers are not always reliable in the presence of giant platelets or red blood cell fragments. As DIC is complex with variable clinical expression and therapy needs to be individualized, our paper can be of value in guiding therapy. Larger studies need to be conducted with the clinical picture in mind to evaluate the effectiveness of the predicted DIC score.

## Figures and Tables

**Table 1:** Conditions associated with DIC.

Associated condition	Number of patients
Sepsis	26
Trauma	6
Severe hepatic failure	12
Massive blood loss	3
After surgery	2
Leukemia	1

**Table 2:** ANOVA table for APTT.

	Degrees of freedom	Sum of squares	Mean squares	*F*	Significance *F*
Regression	1	7.083	7.083	2.859	0.097
Residual	48	118.917	2.477		

Total	49	126			

**Table 3:** ANOVA table for PT/APTT.

	Degrees of freedom	Sum of squares	Mean squares	*F*	Significance *F*
Regression	1	13.417	13.417	5.720	0.020
Residual	48	112.582	2.345		

Total	49	126			

**Table 4:** ANOVA table for PT.

	Degrees of freedom	Sum of squares	Mean squares	*F*	Significance *F*
Regression	1	13.48	13.48	5.75	0.02
Residual	48	112.51	2.34		

Total	49	126			

**Table 5:** ANOVA table for PT + APTT.

	Degrees of freedom	Sum of squares	Mean squares	*F*	Significance *F*
Regression	1	11.51	11.51	4.82	0.03
Residual	48	114.48	2.38		

Total	49	126			

**Table 6:** Predicted DIC scores for the ISTH score of 4. Mean values for predicted scores are 3.60, 3.57, and 3.67.

ISTH score	Predicted score from PT	Predicted score from PT + APTT	Predicted score from PT/APTT
4	3.59	3.47	4.12
4	3.72	3.61	4.09
4	3.55	3.72	3.08
4	4.34	4.37	3.79
4	3.46	3.45	3.43
4	4.10	3.96	4.21
4	3.44	3.56	3.05
4	3.48	3.43	3.70
4	3.40	3.43	3.19
4	3.44	3.43	3.43
4	3.72	3.60	4.12
4	3.53	3.48	3.70
4	3.40	3.35	3.55
4	3.44	3.36	3.82
4	3.57	3.51	3.76
4	3.55	3.50	3.73

**Table 7:** Predicted DIC scores for ISTH score of 5. Mean values for predicted DIC scores are 3.57, 3.64, and 3.72.

ISTH score	Predicted score from PT	Predicted score from PT + APTT	Predicted score from PT/APTT
5	3.44	3.56	3.05
5	3.42	3.38	3.52
5	3.53	3.41	4.12
5	3.57	3.47	3.96
5	3.44	3.42	3.49
5	3.53	4.71	2.45
5	4.21	3.82	5.57
5	3.46	3.41	3.67

**Table 8:** Predicted DIC scores for ISTH score of 3. Mean predicted values are 3.52, 3.69, and 3.38.

ISTH score	Predicted score from PT	Predicted score from PT + APTT	Predicted score from PT/APTT
3	3.38	3.36	3.35
3	3.42	3.37	3.55
3	3.65	3.52	4.21
3	3.65	4.19	2.81
3	3.76	4.80	2.63
3	3.40	3.36	3.52
3	3.36	3.32	3.40
3	3.61	3.61	3.58

**Table 9:** Predicted DIC score for ISTH score of 2. Mean predicted scores are 3.38, 3.35, and 3.37.

ISTH score	Predicted score from PT	Predicted score from PT + APTT	Predicted score from PT/APTT
2	3.44	3.37	3.70
2	3.48	3.49	3.43
2	3.34	3.31	3.29
2	3.34	3.31	3.29
2	3.36	3.32	3.40
2	3.34	3.31	3.29
2	3.38	3.39	3.23

**Table 10:** Predicted DIC score for ISTH score of 6. Mean predicted scores are 4.10, 3.93, and 3.92.

ISTH score	Predicted score from PT	Predicted score from PT + APTT	Predicted score from PT/APTT
6	3.48	3.41	3.79
6	3.51	3.58	3.23
6	6.94	6.09	5.09
6	3.55	3.48	3.82
6	3.55	3.49	3.76
6	3.61	3.54	3.88

**Table 11:** Predicted DIC score for ISTH score of 1. Mean predicted scores are 3.42, 3.37, and 3.58.

ISTH score	Predicted score from PT	Predicted score from PT + APTT	Predicted score from PT/APTT
1	3.38	3.33	3.49
1	3.46	3.41	3.67

**Table 12:** Predicted DIC score for ISTH score of 0. Mean predicted scores are 3.35, 3.32, and 3.31.

ISTH score	Predicted score from PT	Predicted score from PT + APTT	Predicted score from PT/APTT
0	3.34	3.31	3.29
0	3.34	3.31	3.29
0	3.38	3.36	3.37
